# Trimester-specific reference ranges for thyroid hormones of pregnant females at tertiary care hospitals in Lahore, Pakistan

**DOI:** 10.1186/s12884-021-04200-x

**Published:** 2021-10-26

**Authors:** Asim Mumtaz, Fauzia Sadiq, Saima Zaki, Hijab Batool, Muhammad Ibrahim, Muhammad Khurram, Usman Ayub Awan, Kiran Saeed, Muhammad Sohail Afzal

**Affiliations:** 1 Department of Pathology Akhtar Saeed Medical College, Lahore, Pakistan; 2 Lahore Medical & Dental College, Lahore, Pakistan; 3 Department of Gynae & OBS Jinnah Hospital Lahore, Lahore, Pakistan; 4 Central Park Medical College, Lahore, Pakistan; 5 Department of Biostatistics, School of Alibied Health Sciences, Children Hospital Lahore, Lahore, Pakistan; 6 Department of Life Sciences, School of Science, University of Management and Technology (UMT), Lahore, Postal Code # 54700 Pakistan; 7 Department of Medical Laboratory Technology, The University of Haripur, Haripur, Khyber Pakhtunkhwa Pakistan; 8 University of Lahore, Lahore, Pakistan

**Keywords:** Pakistan, Pregnancy, Reference Range, Thyroid function test, TSH, FT_3_, FT_4_

## Abstract

**Background:**

The significance of investigation for diagnosing and managing thyroid dysfunction in pregnant females has been extensively documented in the medical literature. This study aimed to determine trimester-specific reference ranges for thyroid-stimulating hormones (TSH), free T_3_ (FT_3_), and free T_4_ (FT_4_) in apparently healthy pregnant women attending tertiary care hospitals in Lahore.

**Methods:**

This cross-sectional study was conducted at two tertiary care Hospitals in Lahore, Pakistan. In this multi-centric study, 500 pregnant females were initially enrolled from September 2019 to December 2019 who fulfilled the inclusion criteria. For measurement of serum FT_3_, FT_4_, thyroid stimulating hormone (TSH), anti-thyroid peroxidase (anti-TPO), and thyroglobulin antibodies, 5 ml of the blood sample was drawn, under aseptic conditions, from each subject using Maglumi 800 chemiluminescence immunoassay (CLIA) system.

**Results:**

Out of 500 subjects, 23 subjects with positive anti-TPO, 19 subjects with anti-TG antibodies, and 12 subjects due to less volume of serum yielded from whole blood (serum less than 3 ml) were excluded from the analysis. Ten samples were hemolyzed and not included in the analysis. A total of 436 samples were examined for analysis. Of the remaining 436 subjects, 133 (30.5%) were from 1st trimester, 153 (35.1%) from 2nd trimester, and 150 (34.4%) from 3rd trimester. As the data were non-normal, the 2.5th, 50th, and 97.5th percentiles were calculated to express each group's results. Trimester specific range of TSH 0.168-4.294, 0.258-4.584 and 0.341-4.625 mIU/mL, FT_3_1.857-4.408, 1.958-4.621 and 2.025-4.821 pmol/L and FT_4_ 8.815-18.006, 8.306-17.341 and 7.402-17.292 pmol/L.

**Conclusion:**

In this study, we established a trimester-specific reference range for our local population's thyroid function test. The results of this study have complemented the results of previous studies.

## Background

Pregnancy is a physiological phenomenon in which there is the maternal adjustment of multiple organ systems, including metabolic and hormonal adjustments, to supply adequate nutrition to the fetus [1, 2]. During this process, the thyroid gland adapts through regulating thyroid hormones via the hypothalamic-pituitary-thyroid axis [3]. Thyroid hormones are necessary to ensure the healthy development of the fetus, especially during the first trimester, during which the fetus is entirely dependent on the maternal thyroid supply delivered through the placenta [4]. Moreover, during pregnancy, there is increased maternal renal iodide loss, increased levels of serum total thyroxine-binding globulin (TBGs), and increased degradation of thyroid hormone by placental enzymes [5]. Human chorionic gonadotropin (hCG) has a striking structural resemblance with thyroid-stimulating hormone (TSH), leading to an increase in thyroid hormone production during pregnancy followed by a plateau phase around 16 weeks of gestation [6].

Thyroid gland dysfunction is encountered commonly during pregnancy and has an association with obstetric complications. Most frequently, the thyroid disorder encountered in pregnancy is maternal hypothyroidism [7]. This condition is associated with fetal loss, increased maternal blood pressure, preterm labor, and the child's abnormal mental development subsequently [8]. The prevalence of hyperthyroidism in pregnant women is 0.1-0.4%. On the other hand, around 3% of pregnant women are hypothyroid, of whom 0.5% have overt hypothyroidism and 2.5% present with subclinical hypothyroidism [9]. Almost 10 % of the women their reproductive age are positive for thyroid antibodies. Even without any detectable thyroid dysfunction, the presence of anti-thyroid peroxidase antibodies in pregnant women increases the risk of miscarriage, pregnancy-related complications, and preterm labor [10–12]. Gestational thyrotoxicosis is frequently reported in the Asian population compared to western women and pregnant females belonging to other ethnicities [13]. There is a wide geographical variation in the prevalence of thyroid disorders during pregnancy. Therefore, International Endocrine associations advised that each geographical area should establish its trimester-specific reference ranges for thyroid profile. These population-specific reference ranges will aid in the early detection of thyroid abnormalities in pregnant females and will ultimately prevent complications [6, 13, 14].

During pregnancy, thyroid status assessment is critical for initiating treatment in newly diagnosed individuals and adjusting doses in those currently on hormone therapy [15]. The upper and lower TSH limits were reduced relative to the healthy non-pregnant population in pregnant females [16]. In clinical practice, free T4 (FT_4_) and TSH measurements, particularly the cut-off values for lower FT_4_ and higher TSH reference limits, give data for assessing thyroid function in populations [1].

There is currently a dearth of data on the reference values for thyroid indicators in pregnant females in the Pakistani community. The purpose of this study is to establish trimester-specific reference ranges for TSH, free T_3_ (FT_3_), and FT_4_ in apparently healthy pregnant women and are admitted to tertiary care hospitals in Lahore.

## Methods

### Participants

A multi-centric, cross-sectional population survey was conducted at two tertiary care Hospitals in Lahore, Pakistan. A total of 500 pregnant females in all three trimesters of gestation who fulfilled the selection criteria were included in the study using a consecutive sampling technique. Cases with a history of thyroid disease and the use of any thyroid medication were excluded from the study. The study's purpose and procedure were explained to each woman and informed written consent was obtained. Patient details were added on a pre-designed proforma. The gestational age of each case was recorded according to the latest ultrasound scan. First, second and third trimester were defined as gestational age of < 12 weeks, 12-24 weeks and > 24 weeks, respectively. Ethical approval was obtained from institutional review boards of Central Park Medical College, Jinnah Hospital, and Lahore Medical & Dental College (LMDC). Sample collection was carried out between September 2019 and December 2019 after the grant of written permission from concerned authorities of Central Park Teaching Hospital, Jinnah hospital Lahore and Ghurki Trust Hospital.

### Laboratory methods

Five ml of the blood sample was drawn, under aseptic conditions, from each subject to measure their serum FT_3_, FT_4_, TSH, anti-thyroid peroxidase (anti-TPO), and Thyroglobulin antibodies. The samples were labeled and centrifuged within a half-hour. Each patient's serum was transferred to sterilized Eppendorf 05 ml screw-cap tubes and transported to the Pathology lab of Central Park Medical College Lahore in proper sample transport containers with ice bags daily. The samples which were not analyzed immediately were stored at -20 °C. Measurement of serum FT_3_, FT_4_, TSH, anti-TPO, and Thyroglobulin antibodies were performed on Maglumi 800 chemiluminescence immunoassay (CLIA) system. The tests were carried out in batches after collection. However, for the interpretation purpose the laboratory values taken for different parameters were as follows: FT_4_ = 0.89–1.76 ng/dL (11.5–22.7 pmol/L), FT_3_ = 1.8–4.2 pg/mL (2.76–6.45 pmol/L), TSH = 0.3-4.5 mIU/mL, anti-TPO and anti-Tg levels above ≥35 IU/mL and > 40 IU/mL were considered elevated.

### Data analysis

The results were entered and analyzed using Statistical Package for the Social Sciences (SPSS) version 23. The data were tested for normality using the Shapiro-Wilk test. Mean ± SD, median 2.5th, 97.5th percentiles for reference interval were calculated for TSH, FT_3_, and FT_4_ for each trimester. Kruskal-Willis test (a non-parametric test to compare more than three measures) was used to compare the statistic. A *p-value* < 0.05 was taken as statistical significance. A histogram of each thyroid function was also made to have a view regarding the normality of data.

## Results

A total of 500 pregnant females were enrolled in this study. Data of pregnant females with positive anti-TPO antibodies (*n* = 23) and anti-TG antibodies (*n* = 19) were excluded and not analyzed. Samples of twelve pregnant females were not included because the quantity yielded from whole blood was less than 3 ml in quantity. Ten samples were hemolyzed and were not included in the study. A total of 436 samples were included in the study, and out of these, 133 (30.5%) females were in the first trimester, 153 (35.1%) were in the second trimester, and 150 (34.4%) in the third trimester of pregnancy.

**Fig. 1:**
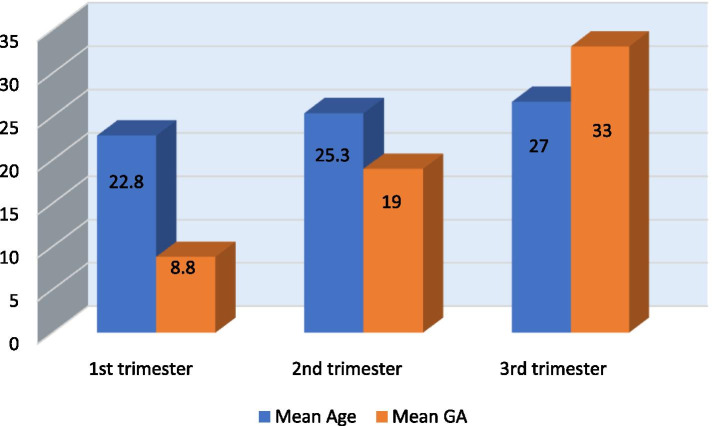
Bar charts of age and GA of study subjects

**Fig. 2:**
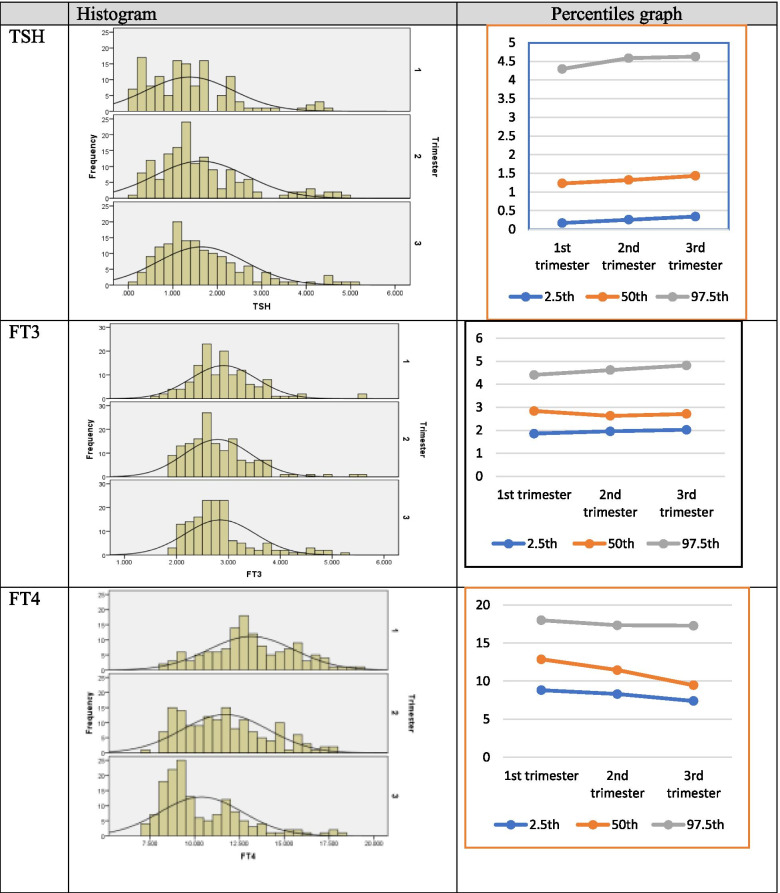
The pattern of trimester specific range of thyroid function TSH, FT3, FT4

The mean age of all females was 25.03 ± 4.06 (with the range 18-35 years). However, Table 1 summarizes the trimester-wise mean age and gestational age of the study patients. The average gestational age 8.79 ± 2.15 (range 4-12 weeks), 18.94 ± 3.06 (13-24 weeks), and 33.06 ± 3.13 (25-39 weeks) at the first, second, and third trimester, respectively. The trimester-specific reference range for thyroid hormones of the data of this study is displayed in Table 2. The thyroid function measurement 2.5th, median and 97.5th percentiles are shown in Fig. 1, demonstrating the trend in all trimesters. The results revealed that the TSH value jumped 0.168-4.294 mIU/mL to 0.341-4.625 mIU/mL from 1st trimester to 3rd trimester, the difference found statistically significant (p-value = 0.018). The FT_3_ values also showed an increasing pattern of 1.857-4.408 pmol/L to 2.025-4.821 pmol/L from 1st trimester to 3rd trimester, and the difference is statistically significant (p-value< 0.001). A decline is observed in FT_4_ 8.815-18.006 pmol/L to 7.402-17.292 pmol/L from 1st trimester to 3rd trimester, but the difference is statistically insignificant (p-value = 0.086), as shown in Fig. 2.

**Table 1:** Age and GA distribution of study subjects

Pregnancy	n	Age (Years) Mean ± SD (Min—Max)	Gestational Age (Weeks) Mean ± SD (Min—Max)
1^st^ trimester	133	22.79 ± 3.23 (18-34)	8.79 ± 2.15 (4-12)
2^nd^ trimester	153	25.36 ± 3.90 (18-35)	18.94 ± 3.06 (13-24)
3^rd^ trimester	150	26.67 ± 4.01 (19-35)	33.06 ± 3.13 (25-39)
Overall	436	25.03 ± 4.06 (18-35)	20.70 ± 10.26 (4-39)

**Table 2:** Trimester specific reference ranges of thyroid function TSH, FT3, FT4

Trimester	n	Minimum	Maximum	Mean ± SD	Percentiles		
					2.5^th^	50^th^ (Median)	97.5th
**TSH mIU/mL**							
First	133	0.140	4.575	1.376 ± 0.982	0.168	1.229	4.294
Second	153	0.185	4.922	1.623 ± 1.041	0.258	1.322	4.584
Third	150	0.194	5.016	1.644 ± 0.994	0.341	1.433	4.625
**FT3 pmol/L**							
First	133	1.533	5.590	2.906 ± 0.636	1.857	2.842	4.408
Second	153	1.854	5.593	2.791 ± 0.646	1.958	2.631	4.621
Third	150	1.964	5.182	2.842 ± 0.674	2.025	2.713	4.821
**FT4 pmol/L**							
First	133	8.137	19.349	13.152 ± 2.391	8.815	12.876	18.006
Second	153	7.417	17.561	11.703 ± 2.404	8.306	11.450	17.341
Third	150	7.057	18.201	10.365 ± 2.326	7.402	9.463	17.292

## Discussion

Pakistan is an Iodine deficient country, and pregnant females are at a higher risk of developing iodine deficiency-related thyroid disorders [17]. Distinguishing factors leading to an iodine-deficient state of the population are lack of awareness, myths relating to the use of iodized salts, and difficult accessibility to supplementation [18]. A recent Pakistani study revealed that 29 % of the pregnant females had thyroid disorders, and 8% of the study population had hypothyroidism [16]. Most international studies related to thyroid hormone reference ranges are conducted in Iodine sufficient areas and poorly depict the intervals in our geographical region. International Guidelines recommend screening for thyroid status before conception and during pregnancy as well [19]. A successful screening program depends upon an accurate population-specific reference interval. The most common cause of maternal hypothyroidism in iodine-rich areas is Hashimoto's thyroiditis, whereas, in iodine-deficient areas like Pakistan, it is iodine deficiency [20]*.* A study conducted by Elahi et al. in Lahore, Pakistan, revealed that around 79% of the pregnant females were iodine deficient, and 31% had goiter [16, 21].

Many studies have reported trimester specific reference ranges during pregnancy. However, these reference ranges vary due to differences in ethnic origin, sample size, iodine sufficiency in the population, sample size, and immunoassay choice [22]. Different analytical assays can vary in terms of sensitivity and specificity and can lead to the variable result of the same sample. Reference values must be gestational age-specific, and the testing method must be certified [23]. In the current study, reference ranges for thyroid hormones were defined by 2.5th and 97.5th percentile after excluding anti-thyroid antibodies positive cases. The National Academy of Clinical Biochemistry (NACB) stated that the determination of reference intervals in the study population should be based on specific, well-defined exclusion criteria [24].

In Pakistan, trimester specific reference intervals for thyroid profile must be established for early diagnosis and treatment of maternal thyroid dysfunctions. The correct interpretation of thyroid function tests during pregnancy is of utmost importance and is only possible when calculating reference intervals in a healthy pregnant population free of major interfering factors. Many studies reveal that common factors that affect the thyroid hormone levels during pregnancy are anti-thyroid antibodies, use of thyroid interfering drugs, medication, maternal age, and gestational age.

Derakhshan et al. [25] concluded in their study that reference ranges of thyroid-stimulating hormone differed significantly according to the exclusion of anti-thyroid antibodies. However, the reference ranges of total and free thyroid hormones did not significantly change after excluding pregnant females with positive anti-thyroid antibodies. According to the study, as mentioned above, smoking also affected thyroid hormone levels among pregnant females. Smokers and females highly exposed to cigarette smoke had a lower TSH and higher T3 (both total and free hormone) than those who were not exposed. Various studies have been carried out in different countries to establish reference ranges for thyroid hormones (Table 3) [26–29]. This study's major limitation was that the study population mainly belonged to one ethnicity only.

**Table 3:** Reference ranges of TSH across different trimesters: Comparison of different studies

Country	Year	Number of Patients (n=)	1^st^ Trimester	2^nd^ Trimester	3^rd^ Trimester	Authors
Iran	2005	229	1.71 + 1.38	1.89 ± 1.24	2.12 + 0.77	Zarghami *et al*. [26]
India	2008	541	0.6 - 5	0.435 - 5.78	0.74 - 5.7	Marwaha *et al*. [27]
China	2013	2743	0.06 - 3.13	0.07 - 4.13	0.15 – 5.02	Zhang *et al*. [28]
Pakistan	2008	384	0.05 - 2.8	0.16 - 3.3	NA	Gilani *et al*. [29]
Current study			1.376 ± 0.982	1.62 ± 1.04	1.64 ± 0.99	
			0.16 - 0.29	0.258 - 4.58	0.34 - 4.62	

However, discrepancies in reported results are noticed because of using different assays; irregularities in pregnancy are associated with demographic, environmental, and genetic factors.

A study conducted in Greece stated that median TSH and FT_3_ increased in the second trimester while FT_4_ levels fell with the advancement of gestational age. Also, there was a significant difference between trimesters' specific FT_4_ values, while no difference was found between TSH and FT_3_ values according to different trimesters [26–28, 30]. These findings justify the segregation of thyroid profile reference intervals into different trimesters.

Data from the Pakistani population reveal that the prevalence rate of subclinical hypothyroidism is around 5.8% and is more common among pregnant females [19]. The Asian population has a greater incidence of thyrotoxicosis as compared to the European community [15, 31]. However, to accurately assess the pregnant females' thyroid status and make correct medical decisions, a population-specific reference interval is necessary. Our current health care practices do not emphasize routine thyroid function screening before and during pregnancy; instead, only high-risk females are screened as a protocol. With the establishment of Trimester specific reference intervals, women with pregnancy-induced thyroid disorders can be accurately managed, and disease burden can be determined.

## Conclusion

This study established a trimester-specific reference range for thyroid function tests for our local population. The results of this study have complemented the results of previous studies. This study will help create the importance of reference ranges of thyroid profile in pregnant females and help the gynecologist, pathologist, and patients interpret TSH, FT_4_ and FT_3_ results because of reference ranges per trimester. It can further help in decreasing maternal morbidity and mortality levels, along with fetal complications in Pakistan.

## Data Availability

The datasets used and analyzed during the current study are available from the corresponding author on reasonable request.

## Abbreviations

TSH: Thyroid-stimulating hormones
FT_3_: Free T_3_
FT_4_: Free T_4_
CLIA: Chemiluminescence immunoassay
hCG: Human chorionic gonadotropin
TBGs: Thyroxine-binding globulin
LMDC: Lahore medical & dental college
SPSS: Statistical package for the social sciences
NACB: National academy of clinical biochemistry
